# The use of lateral arthroscopy portals for the management of bilateral osteochondritis dissecans of the radial head in an English bulldog

**DOI:** 10.1111/vsu.13874

**Published:** 2022-09-02

**Authors:** Samantha E. Shetler, Valentine D. Verpaalen, Whitney D. Hinson, Mélissa De Lombaert, Stephanie A. Belhorn, Robson F. Giglio

**Affiliations:** ^1^ Department of Small Animal Medicine and Surgery, College of Veterinary Medicine University of Georgia Athens Georgia

## Abstract

**Objective:**

To report a case of bilateral radial head osteochondritis dissecans (OCD) in a dog treated via lateral elbow arthroscopy portals.

**Study design:**

Case report.

**Animals:**

Six month old female spayed English bulldog.

**Methods:**

The dog was presented for a left thoracic limb lameness localized to the elbow. Computed tomography revealed bilaterally symmetrical mineralized fragments in the lateral compartment of the elbow joint and blunting of the medial coronoid processes. The fragments were associated with a thin donation bed along the caudolateral articular surface of the radial head with moderate surrounding subchondral bone sclerosis. Bilateral elbow arthroscopy was pursued. Arthroscopy was initiated via a standard medial approach, which allowed for abrasion arthroplasty of the radial incisure and medial coronoid process but provided insufficient access to the radial head lesions. A lateral arthroscopic approach was subsequently performed and provided excellent access to the radial head for fragment retrieval and abrasion arthroplasty.

**Results:**

Histopathology of the radial head fragments revealed mild cartilage degeneration and retention of cartilaginous cores within subchondral bone, consistent with OCD. Complete resolution of lameness and elbow pain were observed on clinical examination 5 months postoperatively.

**Conclusion:**

Radial head OCD can occur as a rare component of elbow dysplasia in growing dogs, and fragment retrieval with abrasion arthroplasty via lateral arthroscopic portals may be an effective treatment option.

## 1 INTRODUCTION

Osteochondrosis is a developmental disorder resulting from a focal disruption of the normal endochondral ossification process, leading to thickening and retention of cartilage within subchondral bone.^1^ Progressive chondromalacia can subsequently result in fissures that extend from the subchondral bone to the articular cartilage surface, creating a cartilaginous flap. Osteochondrosis in combination with the presence of a cartilaginous flap is further defined as osteochondritis dissecans (OCD) and is an important differential for lameness in juvenile dogs.

Within the canine elbow joint, osteochondrosis of the humeral condyle has been widely reported.^2^, ^3^ Although fragmentation of the medial coronoid process, ununited anconeal process, and ununited medial humeral epicondyle have all been proposed to be manifestations of osteochondrosis, the definitive etiopathogeneses of these conditions remain subject to heated debate.^4^, ^5^, ^6^, ^7^, ^8^, ^9^ As far as the authors are aware, osteochondrosis of the radial head has not yet been described in animals. Radial head OCD has been described in humans, although it is considered extremely rare.^10^, ^11^, ^12^

Humeral trochlear OCD is typically treated with flap removal and abrasion arthroplasty via a medial arthroscopic approach, although the use of cartilage resurfacing techniques is becoming more common.^13^ The standard medial arthroscopic approach allows for excellent visualization and exploration of the medial compartment, but provides limited access to the cranial compartment, caudal olecranon, and caudolateral compartment.^14^ Although caudal and lateral arthroscopy portals have previously been described in an anatomic canine cadaveric study, as far as the authors are aware, they have not yet been reported in a clinical setting.^15^

As such, this case report describes the clinical presentation, diagnostic findings, and clinical outcome of radial head OCD in a dog treated by flap excision and abrasion arthroplasty using lateral arthroscopy portals.

## 2 MATERIALS AND METHODS

**FIGURE 1:**
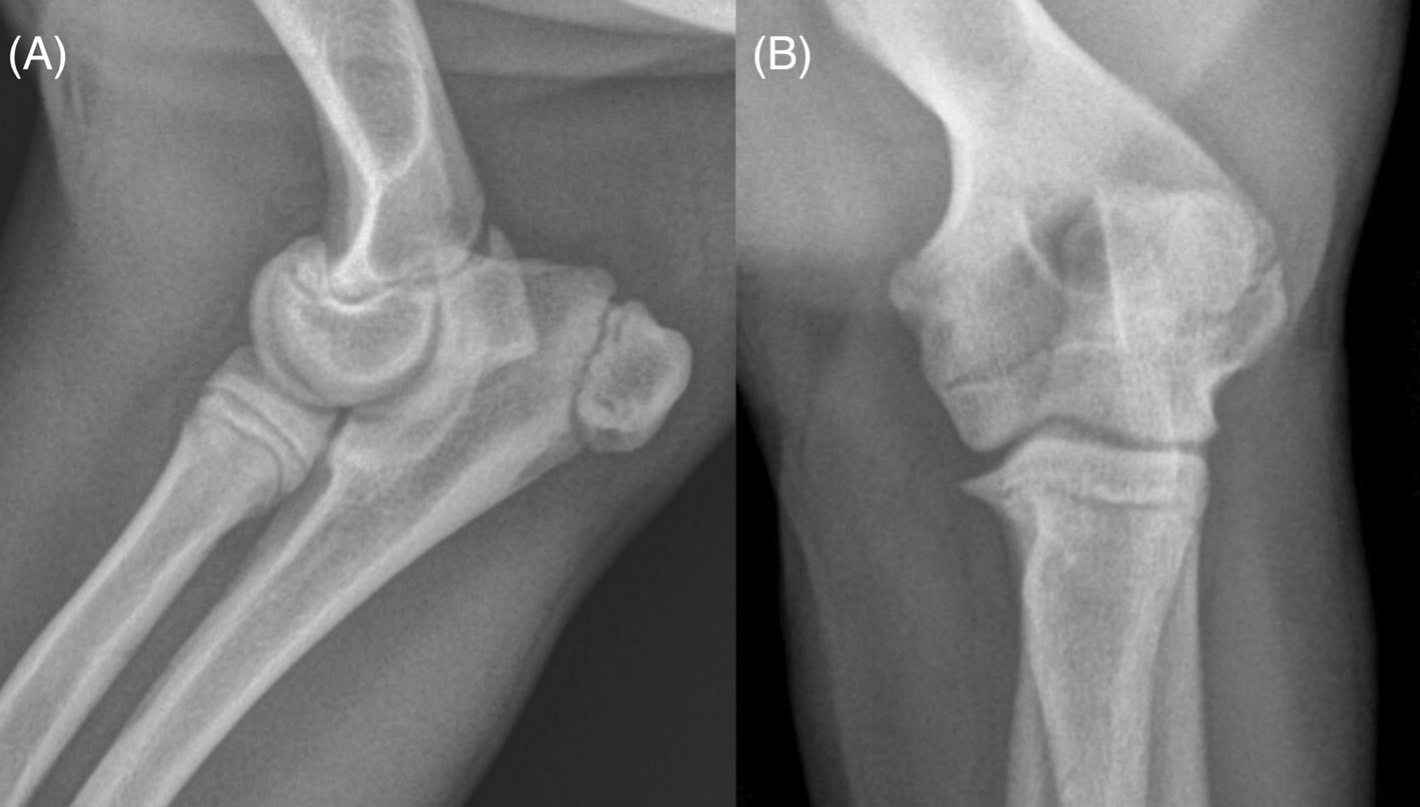
Lateral (A) and craniocaudal (B) radiographs of the left elbow. No abnormalities of the osseous and soft tissue structures are identified

A 6 month old female spayed English bulldog weighing 17.2 kg was presented to the University of Georgia Veterinary Teaching Hospital for evaluation of a persistent left thoracic limb lameness of 2 months’ duration. The onset of lameness was acute, with no traumatic event witnessed. Radiographs of the left elbow were performed by the primary veterinarian at onset of lameness and were unremarkable (Figure 1). A brief trial with a nonsteroidal anti‐inflammatory drug was performed and did not result in any improvement.

Orthopedic examination revealed a body condition score of 5 of 9, mild muscle atrophy of the left thoracic limb, and a grade 2 out of 5 left thoracic limb lameness.^16^ At a stance, the left elbow was held in abduction with external rotation of the antebrachium. The left elbow had mild effusion and was painful on extension. The remainder of the orthopedic examination was unremarkable.

**FIGURE 2:**
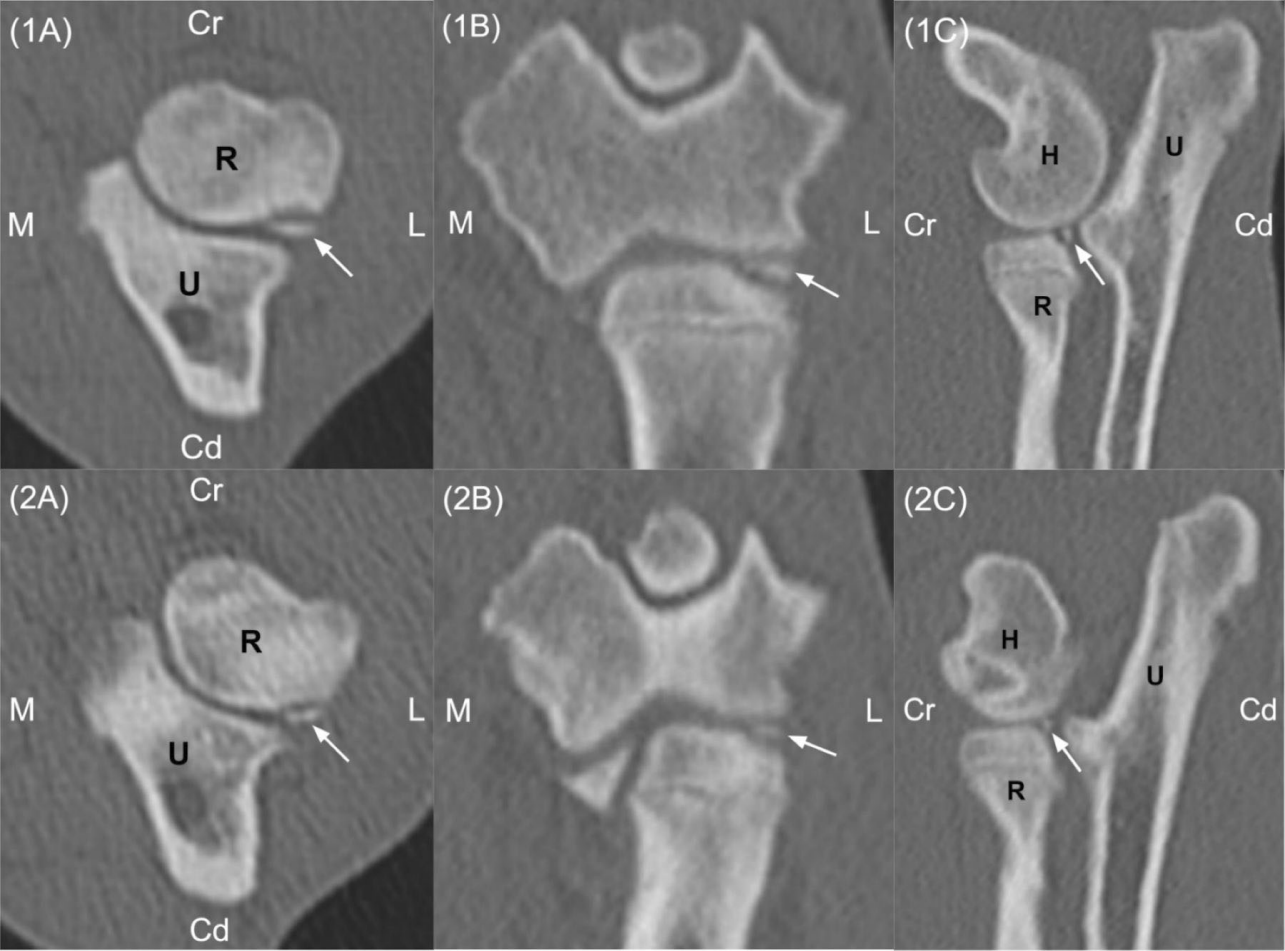
Computed tomography (CT) images of the left (1A‐1C) and right elbows (2A‐2C) in an English bulldog with bilateral radial head osteochondritis dissecans (OCD). Axial (1A, 2A), coronal (1B, 2B), and sagittal (1C, 2C) images in a bone window demonstrate the thin linear OCD fragment (white arrow) and associated subchondral defect with adjacent sclerosis along the caudolateral surface of the radial head. Laterality markers: M (medial), L (lateral), Cr (cranial), and Cd (caudal). Bone labels: R (radius), U (ulna), and H (humerus)

Computed tomography (CT) of both thoracic limbs was performed and revealed a mineral attenuating fragment in the lateral aspect of the proximal radioulnar joint bilaterally, adjacent to the lateral aspect of the radial head (Figure 2). The right mineralized fragment measured 3.1 mm × 0.8 mm × 1.0 mm, and the left fragment measured 4.5 mm × 0.9 mm × 1.7 mm. There was a corresponding thin donation bed along the caudolateral articular surface of the radial head with moderate associated sclerosis of the surrounding subchondral bone. Mild osteophytosis was also present along the lateral coronoid process in the right elbow. Bilaterally, moderate blunting and mild sclerosis of the medial coronoid processes was observed. There was no appreciable humeroulnar or radioulnar incongruity.

**FIGURE 3:**
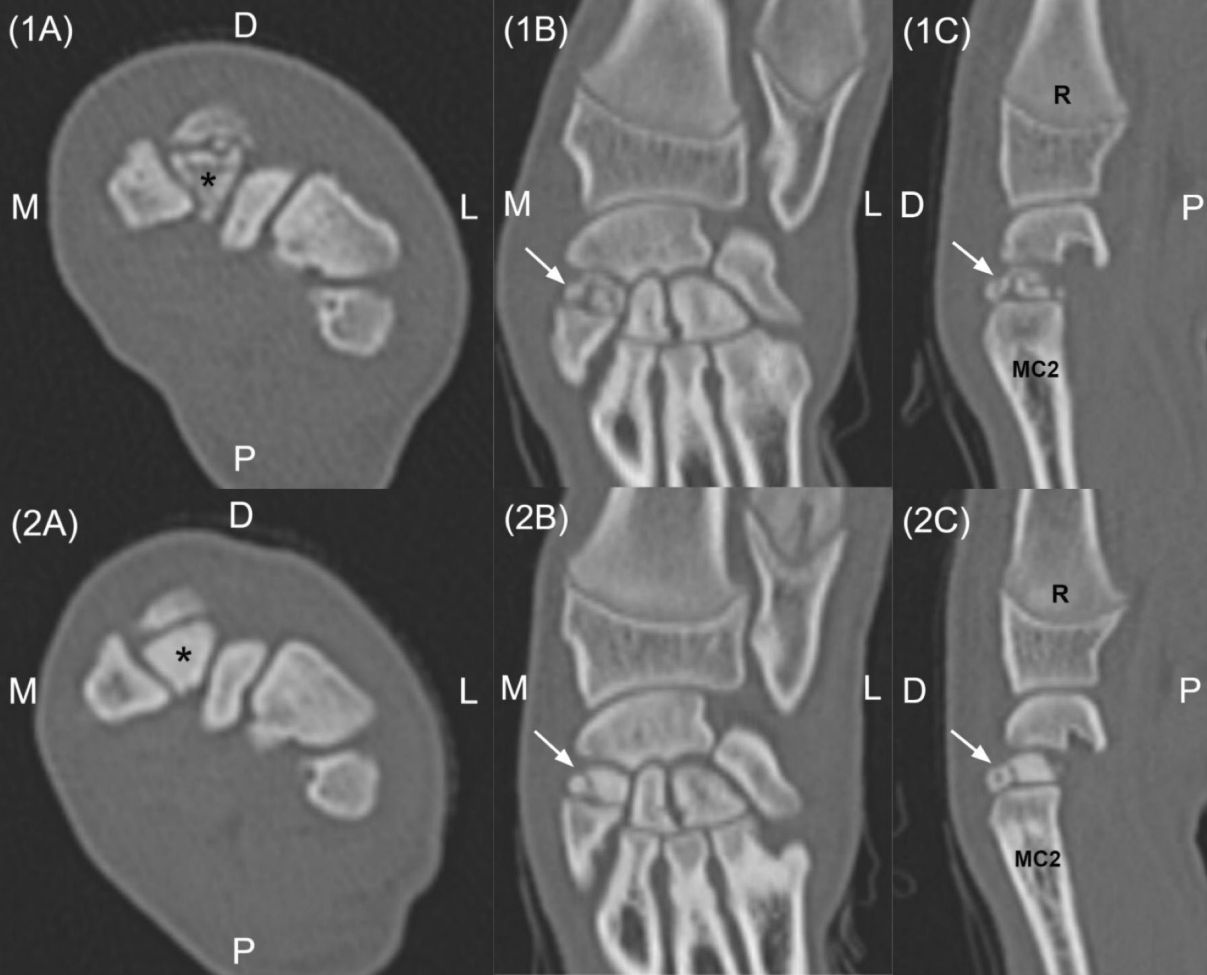
Computed tomography (CT) images of the left (1A‐1C) and right carpus (2A‐2C) in an English bulldog with bilateral multipartite second carpal bones. Axial (1A, 2A), coronal (1B, 2B), and sagittal (1C, 2C) images in a bone window demonstrate fragmentation of the second carpal bone (black asterisk and white arrow). Laterality markers: M (medial), L (lateral), D (dorsal), and P (palmar). Bone labels: R (radius) and MC2 (second metacarpal bone)

In addition to the findings described above, CT revealed fragmentation of the second carpal bones bilaterally (Figure 3). In the right second carpal bone, there were 2 larger fragments with additional mild fragmentation of the medial aspect of the dorsal fragment. The left second carpal bone was more severely affected with extensive fragmentation. All fragments were smoothly marginated and, in the absence of soft tissue swelling, consistent with multipartite second carpal bones. Orthopedic examination of the carpal joints was unremarkable, with no pain elicited on direct palpation of the second carpal bones. Although unique, these findings were therefore concluded to be incidental in nature.

The owners elected to pursue bilateral elbow arthroscopy. The patient was premedicated with hydromorphone (0.1 mg/kg IM), dexmedetomidine (3 mcg/kg IM), and maropitant (1 mg/kg IV). General anesthesia was induced with ketamine (1 mg/kg IV) and propofol (4 mg/kg IV to effect), and maintained with isoflurane inhalation (1%‐2%). Both thoracic limbs were clipped and prepared via the standard technique.

**FIGURE 4:**
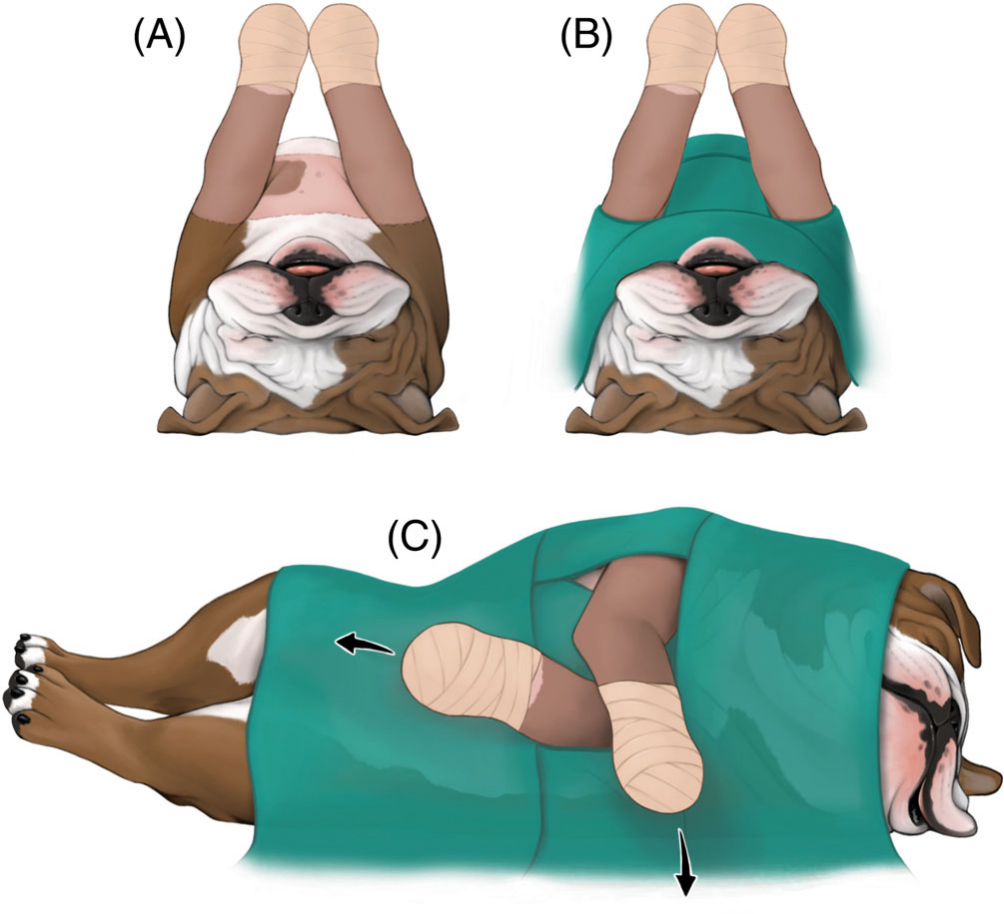
Patient preparation and positioning for the lateral arthroscopic approach. The patient was positioned in dorsal recumbency; both thoracic limbs were aseptically prepared in a hanging limb position and draped routinely (A and B). For the lateral arthroscopic approach, the thoracic limb was positioned in adduction and external rotation while the contralateral limb was pulled caudally in extension (C). Sterile towels were placed underneath the elbow as needed to provide a fulcrum. Note that, as a result of limb manipulation, the head and thorax were repositioned in slight lateral recumbency. Illustration by Madison Christian, Educational Resources, © University of Georgia.

The dog was positioned on the operating table in dorsal recumbency. Both thoracic limbs were aseptically prepared and draped routinely (Figure 4). Sterile towels were placed on either side of the thoracic limbs as fulcrums for joint distraction. Arthroscopy of the left elbow was initiated using a 30°, 1.9 mm arthroscope (Arthrex Inc., Naples, Florida) and standard medial portals.^17^ Although the entire procedure was recorded, unfortunately, the arthroscopic images were not stored due to malfunctioning software. A reconstruction of the arthroscopic findings has therefore been provided using medical illustrations.

**FIGURE 5:**
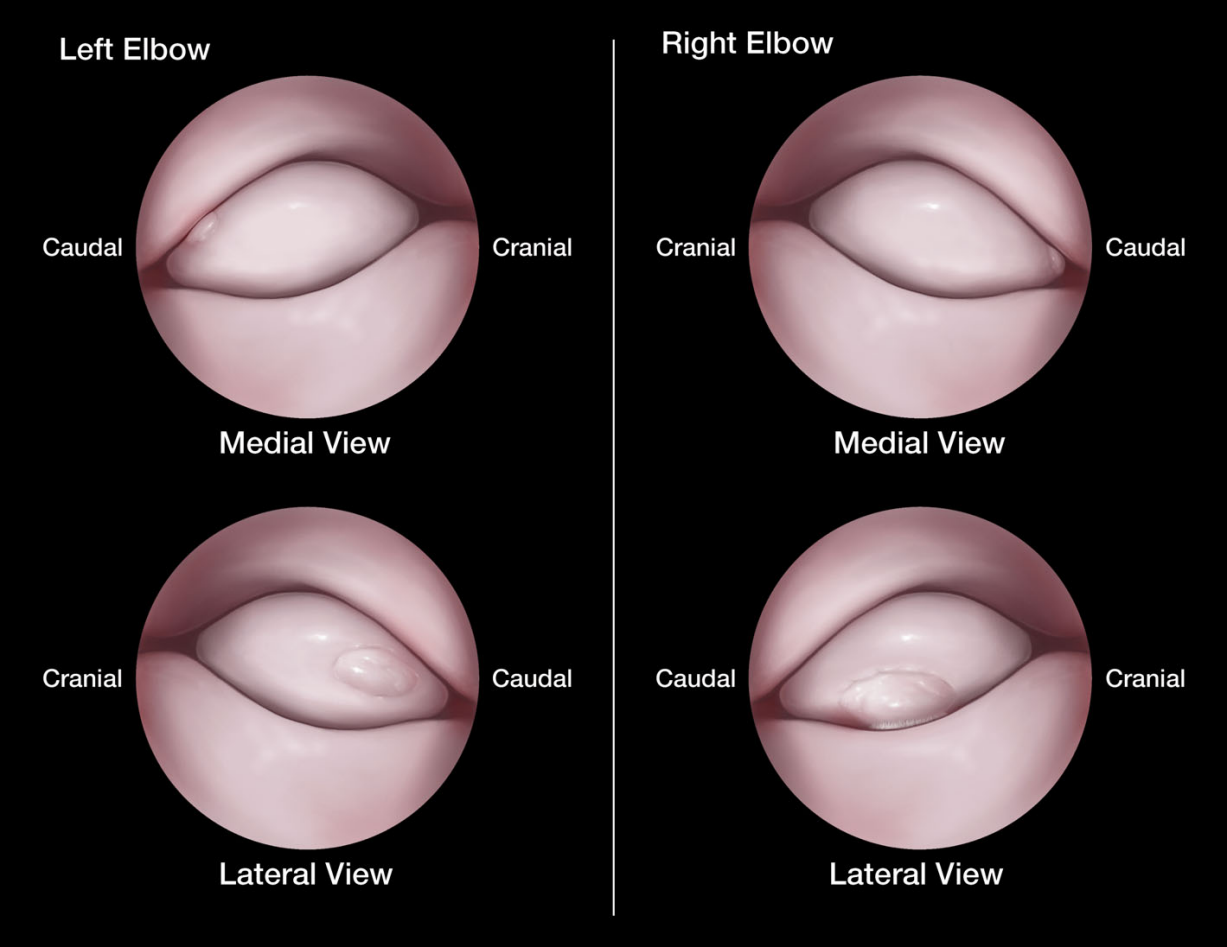
Illustrations depicting the arthroscopic appearance of the radial head lesions. Both osteochondral lesions were located on the caudolateral aspect of the radial head and characterized by discoid elevation of cartilage with an obvious surrounding cleft. The standard medial arthroscopic approach (top images) allowed for partial visualization of the osteochondral lesions but did not provide sufficient access for probing or debridement. A lateral arthroscopic approach (bottom images) was subsequently performed and allowed for complete evaluation and treatment of the osteochondral lesions. The lesion associated with the left radius was slightly less marginalized and extended further along the proximal weight‐bearing surface, which could explain the unilateral left thoracic limb lameness observed in this dog. Fibrous attachments were observed at the distal margin of the lesion associated with the right radius. Treatment consisted of fragment removal and abrasion arthroplasty. Illustration by Madison Christian, Educational Resources, © University of Georgia.

Mild synovitis was noted upon entry of the medial compartment of the left elbow. A radial incisure fissure and blunting of the medial coronoid process were observed. There was a negative radioulnar step of approximately 1 mm, isolated to the tip of the medial coronoid process. Upon gentle probing with the meniscal probe, notable cartilage softening was appreciated on the entire medial coronoid process as well as the adjacent border of the radial head with a Modified Outerbridge Score (MOS) of 1. The remainder of the ulna and the entire humeral condyle were normal. Upon further inspection of the radial head, a cartilage flap was partially visible on the caudolateral articular surface (Figure 5). Probing of the fragment was not feasible through the medial portals. Abrasion arthroplasty of the radial incisure, medial coronoid process, and adjacent diseased portion of the radial head was completed using a small curette. The underlying subchondral bone was soft and discolored. Abnormal subchondral bone was curetted until bleeding, healthy bone was exposed. The arthroscope and instruments were removed and the portals were closed routinely.

**FIGURE 6:**
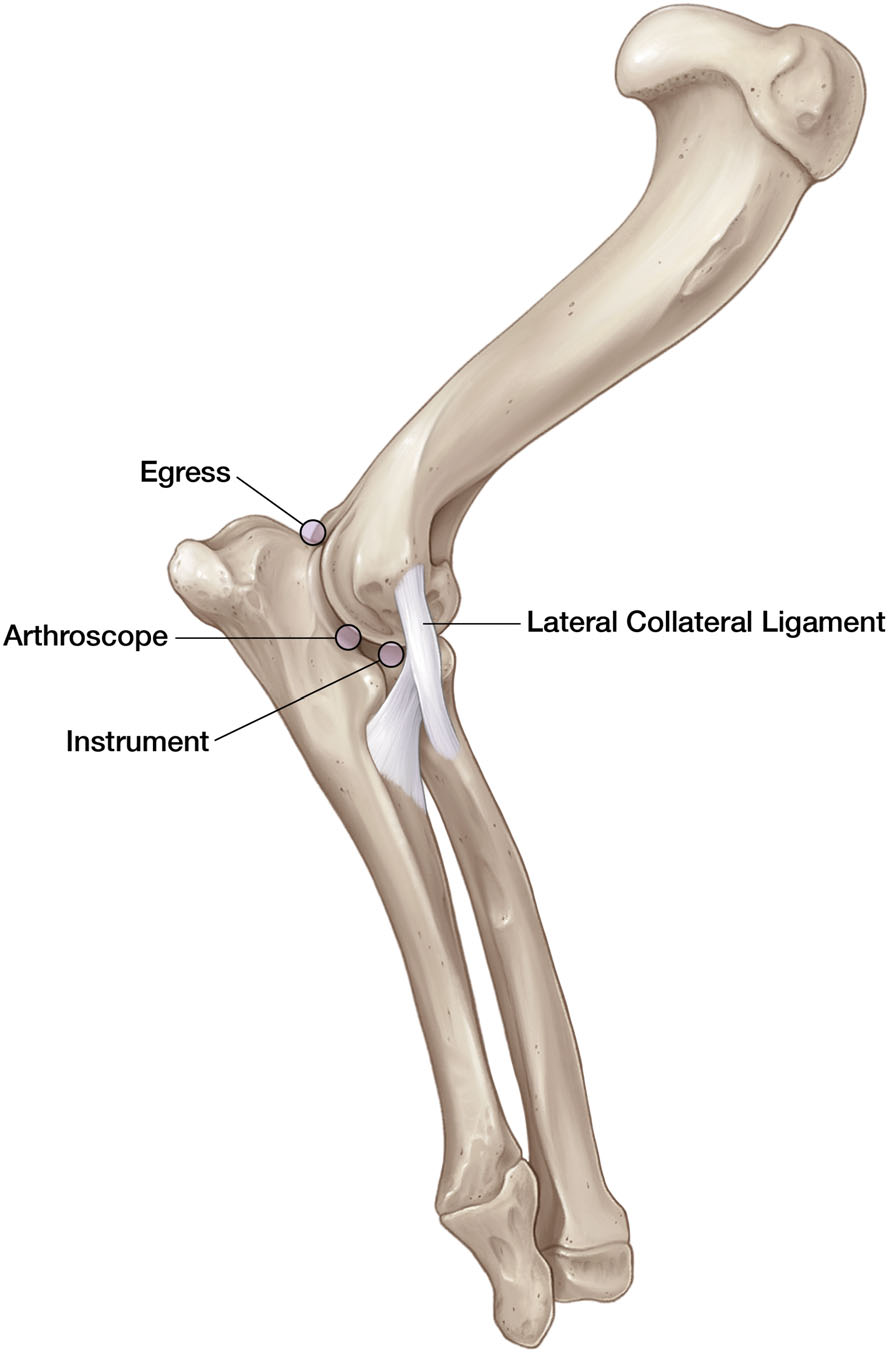
Portal locations and pertinent anatomy for canine elbow arthroscopy via a lateral approach. The arthroscope portal was established in the center of a triangle formed by the lateral epicondyle, radial head, and olecranon tuberosity. Egress was established adjacent to the anconeus in a craniodistal and medial direction into the joint. The craniolateral instrument portal was established between the lateral epicondyle and the radial head approximately 1 cm cranial to the arthroscope portal and caudal to the lateral collateral ligament. Illustration by Madison Christian, Educational Resources, © University of Georgia.

The left thoracic limb was then repositioned in adduction and external rotation to allow for lateral joint exploration (Figure 4). A straight lateral arthroscope portal was established in the center of a triangle formed by the lateral epicondyle, radial head, and olecranon tuberosity (Figure 6).^15^ Egress was established by inserting a 1½ inch, 18 gauge needle adjacent to the anconeus in a craniodistal and medial direction into the joint. The craniolateral instrument portal was established between the lateral epicondyle and the radial head approximately 1 cm cranial to the arthroscope portal and caudal to the lateral collateral ligament.

Upon entry of the lateral compartment of the left elbow joint, the articular cartilage flap associated with the radius was readily apparent (Figure 5). The cartilage flap was located at the caudolateral margin of the radial head and was characterized by discoid elevation of cartilage with an obvious surrounding cleft. Superficial cartilage fibrillation was apparent on the surface of the lateral coronoid process (MOS 2). The cartilage flap was easily elevated off of the radial head by gentle probing at the cleavage plane using a meniscal probe. A 5/0 curette was used to detach the cartilage flap. The fragment was subsequently removed in a single piece using arthroscopic grasper forceps and was submitted for histopathologic analysis. The underlying subchondral bone was soft and discolored. The curette was used to debride the diseased subchondral bone until bleeding occurred and healthy bone was exposed. The portals were closed routinely.

Arthroscopic exploration of the right elbow was performed in a similar fashion, starting with a medial approach. Medial coronoid pathology was nearly identical in appearance to the left elbow (radial incisure fissure, blunted medial coronoid process tip, and a 1 mm negative radioulnar step isolated to the medial coronoid apex). Upon gentle probing with the meniscal probe, notable cartilage softening was appreciated on the entire medial coronoid process (MOS 1). Upon inspection of the radial head, a cartilage flap was partially visualized at the caudolateral aspect (Figure 5). Abrasion arthroplasty of the radial incisure and medial coronoid tip was performed as previously described.

The right thoracic limb was then repositioned and lateral portals were established as previously described for the left elbow. Superficial cartilage fibrillation was apparent on the surface of the lateral coronoid process (MOS 2). The cartilage flap associated with the most caudal aspect of the lateral surface of the radial head was readily apparent (Figure 5). The fragment was slightly more marginalized in comparison with the left side and obtained fibrous attachments to its distolateral margin. The cartilage flap was loosened using a 5/0 curette, removed using arthroscopic grasping forceps and submitted for histopathology. The underlying subchondral bone was soft and discolored and was curetted until bleeding occurred. Once healthy bone was exposed, instruments were removed and portals were closed routinely.

In the initial postoperative period, methadone (0.1 mg/kg IV every 4‐6 hours) and carprofen (2.2 mg/kg IV once) were administered for pain control. The day following surgery, the dog was bearing weight well on both thoracic limbs with no apparent lameness or elbow discomfort. The dog was transitioned to carprofen (2.2 mg/kg PO every 12 hours for 7 days) and trazodone (3 mg/kg PO every 8‐12 hours as needed for activity restriction) and was discharged from the hospital. The owners were instructed to keep her confined to a crate when unsupervised and to restrict activity to short leash walks until her progress evaluation at 4‐6 weeks postoperatively.

## 3 RESULTS

Histopathology of the arthroscopic samples revealed evidence of cartilage degeneration characterized by chondrocyte cloning and decreased basophilia associated with loss of matrices. Islands of retained, degenerate cartilage surrounded by subchondral bone were observed in a focally extensive area of the fragment sampled from the right elbow. These findings were strongly supportive of a diagnosis of OCD.

Due to unforeseen owner circumstances, the dog was not able to be returned for clinical evaluation until 5 months postoperatively. The owners reported that the dog had returned to normal activity levels and demonstrated excellent limb use shortly after surgery. Upon clinical evaluation, the dog exhibited no appreciable lameness or elbow effusion and had full range of motion of both elbows.

## 4 DISCUSSION

This case report documents the use of a lateral arthroscopic approach for the successful treatment of radial head OCD in a dog. Several factors were taken into consideration to justify the diagnosis of radial head OCD, including patient signalment, history, diagnostic imaging, and histopathology. Similar to the dog described in this case report, osteochondrosis most commonly occurs in medium‐to large breed dogs that are in a rapid phase of musculoskeletal growth, around 4 to 8 months of age.^2^, ^3^ Typical CT features of OCD include focal subchondral bone defects with surrounding subchondral sclerosis and the presence of mineral attenuating intra‐articular fragments, all of which were present in the dog described in this case report.^18^ Histopathology of the fragments revealed retained, degenerative cartilage within subchondral bone, which further supports a diagnosis of OCD.^1^ It is important to note that these histologic findings are characteristic of, but not pathognomonic for, osteochondrosis. A definitive diagnosis of OCD is often not possible on the basis of histopathology alone due to the chronicity of the disease at the time of diagnosis. Although traumatic fragmentation could be an important differential for this case, the lack of a history of trauma, the bilateral symmetry, and the unusual location of the lesions are all inconsistent with a single traumatic event.

Arthroscopic examination allowed for further classification of the OCD lesions. In the dog described in this case report, both cartilage flaps occurred at the caudolateral margin of the radial head, and the lesion associated with the right radius obtained obvious fibrous attachments to its distolateral margin. These findings appear most consistent with type 2 osteochondrosis, as previously defined by Olsson.^4^

As far as the authors are aware, this is the first report describing OCD of the radial head in a dog. Radial head OCD has rarely been described in human adolescents, however, and is often associated with radial head subluxation.^10^, ^11^, ^12^ Although the exact etiology remains unclear, the prevailing theory is that these lesions occur secondary to joint incongruity in combination with repetitive throwing activities, which lead to impingement of the posteriomedial aspect of the radial head on the center of the capitellum.^11^ Nonpainful or early stable lesions were managed conservatively, while arthroscopic fragment removal, abrasion arthroplasty, and/or subchondral bone drilling was recommended for unstable lesions with flap detachment or if clinical signs persisted.^10^, ^11^, ^12^ In the case of concomitant radial head subluxation, an oblique ulnar osteotomy with subsequent ulnar distraction and angulation was recommended to reduce the radial head.^11^ Return to full athletic function was achieved in all cases.^10^, ^11^ Given the pathogenesis of radial head OCD in humans, it may be advisable to screen for radial head OCD lesions in dogs diagnosed with radial head luxation or subluxation. It is also important to consider that English bulldogs have a high prevalence for conformation‐related disorders in general, including elbow dysplasia.^19^, ^20^ Although it is possible that English bulldogs may be predisposed to radial head OCD lesions, additional cases are required to substantiate this claim.

In the dog described in this case report, only mild radioulnar incongruity confined to the apex of the medial coronoid process was identified during arthroscopy. This type of incongruity is commonly identified as a component of medial coronoid disease and should not increase stress to the caudolateral aspect of the radial head.^21^ Joint distraction during arthroscopy may complicate the assessment of elbow incongruity, however, care was taken to maintain the elbow in a neutral standing angle, which has been demonstrated to allow for sensitive detection of radioulnar incongruity in a previous study.^22^ Although CT imaging similarly did not reveal any evidence of joint incongruity, it remains plausible that a transient or dynamic incongruity could have occurred, causing repetitive stress to the caudolateral aspect of the radial head. The lesion associated with the left radius was slightly less marginalized and extended further along the proximal weight‐bearing surface of the radius, which could explain the unilateral lameness observed in this dog. Given the concurrent medial coronoid pathology, it is possible that the radial head lesions represented an incidental, subclinical finding. Nevertheless, arthroscopic fragment removal via lateral portals, followed by abrasion arthroplasty, led to a successful outcome in this case.

This case highlights the importance of advanced diagnostic imaging in the diagnosis and treatment of canine elbow disease. As a result of the CT findings, we were able to communicate the unique aspects of this case effectively with the owner preoperatively, and plan appropriately for an alternative approach. The lateral arthroscopic approach was simple to perform and provided excellent access to the lateral humero‐radio‐ulnar compartment with minimal risk of iatrogenic articular cartilage injury. There are multiple potential indications for a lateral arthroscopic approach besides the one described in this case report. Careful evaluation of the lateral compartment is often performed when considering certain surgical treatment options for medial compartment disease, such as the proximal abducting ulnar osteotomy, sliding humeral osteotomy, and canine unicompartmental elbow arthroplasty procedures.^23^, ^24^ Joint evaluation may be better suited through a lateral arthroscopic approach in these cases. An additional benefit of the lateral approach includes the potential for decreased risk of iatrogenic damage to vital anatomic structures due to the absence of major neurovascular bundles, such as the ulnar nerve. As such, a lateral arthroscopic approach may be indicated for the assessment and treatment of generalized elbow joint disease, such as the treatment of septic arthritis, the performance of synovial biopsies, and the assessment of intra‐articular fracture reduction as a component of minimally invasive fracture repair.^15^

Incidentally, CT imaging of this specific case revealed bilateral fragmentation of the second carpal bones. Given the bilateral nature and smooth margination of the fragments, and the lack of associated soft tissue swelling or discomfort on palpation, these lesions most likely represent a congenital or developmental abnormality. As far as the authors are aware, congenital multipartition of the second carpal bone has not been reported previously in dogs.

This case report documents the diagnostic evaluation and successful treatment of radial head OCD in a dog using lateral arthroscopy portals. The lateral arthroscopic approach described in this report was performed easily and uneventfully, and provided excellent access to the lateral compartment of the elbow. Obvious limitations of this report are inherent to the study of a single case. Additional cases are required to obtain further understanding in regards to the etiology, diagnostic features, clinical significance, and optimal management strategies for radial head OCD in dogs.

## CONFLICT OF INTEREST

The authors declare no conflicts of interest related to this report.
